# Prevalence of Opportunistic Infections in Treatment-Naive HIV Patients in a Secondary Care Center

**DOI:** 10.7759/cureus.73297

**Published:** 2024-11-08

**Authors:** Duanie A Moran

**Affiliations:** 1 General Medicine, Hospital General Atlántida, La Ceiba, HND

**Keywords:** antiretroviral naive, cryptococcosis, naïve hiv-infected patients, opportunistic fungal infection, opportunistic infection

## Abstract

Introduction

HIV-related immunosuppression significantly increases the risk of contracting opportunistic infections (OI) due to bacteria, viruses, fungi, and protozoa. This study estimates the prevalence of opportunistic infections in HIV patients who have not received previous antiretroviral therapy (ART) in the second-largest hospital in Honduras.

Methods

This retrospective observational study analyzed the medical records of the treatment-naive HIV patients who attended the Mario Catarino Rivas National Hospital in Honduras in all of 2023. The data collected was on demographics and clinically or laboratorially diagnosed opportunistic infections of 110 patients.

Results

The overall prevalence of OIs among the 110 treatment-naive HIV patients was 31 (28.2%). The most frequent OIs were: cryptococcosis 9/31 (29.0%), candidiasis 8/31 (25.8%), tuberculosis 6/31 (19.4%), toxoplasmosis 4/31 (12.9%), pneumocystis 3/31 (9.7%), and histoplasmosis 2/31 (6.5%). Most patients with at least one OI had a viral load >100,000 copies/mL and a CD4+ cell count <200 cells/mm^3^.

Conclusion

There has been an increase in the prevalence of fungal IOs in treatment-naive HIV patients in Honduras. Adequate allocation of resources for the diagnosis and treatment of these in the country's healthcare system is paramount.

## Introduction

Opportunistic infections (OI) are defined as infections produced with more frequency or severity due to severe immunosuppression [1]. Since the beginning of the HIV epidemic, 42.3 million people have died from AIDS-related illnesses; a large percentage of these are caused by opportunistic infections [2]. HIV-related immunosuppression significantly increases the risk of contracting opportunistic infections due to bacteria, viruses, fungi, and protozoa [3]. The level of immunosuppression, especially with CD4 lymphocytes < 200 cells/mm^3^, is the main risk factor [4]. Although antiretroviral therapy (ART) has significantly decreased the incidence of opportunistic infections, they are still of great importance.

Similar studies have been conducted in other countries. However, in Honduras, the two most recent studies related to opportunistic infections in HIV patients were in 2016 and 2022 [5,6]. The present study aims to fill the knowledge gap found regarding OIs in treatment-naïve HIV patients through a cross-sectional study. It was carried out at the Mario Catarino Rivas National Hospital, the second-largest national hospital and the one with the largest cohort of HIV patients in the country [6]. By calculating the prevalence of OIs and characterizing these patients, the data from this study will be useful for the allocation of resources and will contribute to the knowledge of infectious diseases in this population.

## Materials and methods

Study design and setting

We conducted a retrospective observational study that analyzed the medical records of 110 treatment-naive HIV patients who attended the Mario Catarino Rivas National Hospital, a secondary care center in Honduras, in 2023. To be included in this study, the patients were required to be aged more than 18 years, with no previous ART, have a complete medical record, and have been evaluated by the hospital’s Integrated HIV Service during the study period. Patients who had already received ART or had an incomplete medical record were excluded. 

This study was performed as a graduation project for a master's in HIV from the King Juan Carlos University of Madrid, as part of the Esther Project. The Mario Catarino Rivas National Hospital regularly welcomes researchers from this master's program after reviewing their projects (Appendices 1, 2, 3).

Data collection and statistical analysis

The data collection process involved a comprehensive review of patient records stored at the care center. The patient data recorded included demographic information such as sex, age, educational level, marital status, and employment status. In addition, we recorded information on clinical characteristics such as viral load, CD4 cell count, clinical stage, previous use of pre-exposure prophylaxis (PrEP), and existing comorbidities. Furthermore, we documented the presence of OIs. Data analysis was performed using IBM SPSS Statistics for Windows, Version 28 (Released 2021; IBM Corp., Armonk, New York, United States).

Ethical considerations

The care center provided formal approval, and a confidentiality agreement was signed by the researcher before data collection. No identifiable patient information was collected. The study complied with the Declaration of Helsinki and patient confidentiality.

## Results

We analyzed 110 patients, of which 31 had at least one OI; therefore, the prevalence of opportunistic infections in treatment-naive HIV patients was 28.2% (31/110). The most frequent OIs were: cryptococcosis 9/31 (29.0%), candidiasis 8/31 (25.8%), tuberculosis 6/31 (19.4%), toxoplasmosis 4/31 (12.9%), pneumocystis 3/31 (9.7%), and histoplasmosis 2/31 (6.5%). One patient had both cryptococcosis and candidiasis.

Most patients were male (70.9%), between 20 and 29 years of age (47.3%), single (68.2%), with a complete high school education (23.6%), and employed (68.2%). The sociodemographic characteristics are described in greater detail in Table 1.

**Table 1:** Sociodemographic characteristics of treatment-naive HIV patients at the Mario Catarino Rivas National Hospital, January to December 2023

	Number (n)	Percentage (%)
Sex		
Male	78	70.9
Female	32	29.1
Age		
<20	3	2.7
20-29	52	47.3
30-39	23	20.9
40-49	16	14.5
50-59	14	12.7
≥60	2	1.8
Educational Level		
Illiteracy	2	1.8
Incomplete primary education	17	15.5
Complete primary education	13	11.8
Incomplete secondary education	20	18.2
Complete secondary education	26	23.6
Incomplete higher education	19	17.3
Complete higher education	13	11.8
Marital Status		
Single	75	68.2
Free union	27	24.5
Married	5	4.5
Divorced	2	1.8
Widowed	1	0.9
Employment Status		
Employed	75	68.2
Unemployed	35	31.8

The majority of patients with at least one OI had a viral load >100,000 copies/ml and a CD4+ cell count <200 cells/mm^3^. In this group, the viral load was <1,000 copies/ml in two (6.5%), between 1,000 and 100,000 in eight (25.8%), and >100,000 in 17 (54.8%). Likewise, the CD4+ cell count was <200 cells/mm^3^ in 21 (67.7%), 200-500 cells/mm^3^ in five (16.1%), and >500 cells/mm^3^ in four (12.9%). No reagent was available to perform viral load in four (12.9%) and CD4+ count in one (3.2%) before ART initiation.

In contrast, in the group of 79 patients who did not present OI, the viral load was <1,000 copies/ml in 10 (12.7%), between 1,000 and 100,000 in 50 (63.3%), and >100,000 in 15 (19.0%). Likewise, the distribution of CD4+ cell count was <200 cells/mm^3^ in 11 (13.9%), 200-500 cells/mm^3^ in 39 (49.4%), and >500 cells/mm^3^ in 20 (25.3%). No reagent was available to perform viral load in four (5.1%) and CD4+ count in nine (11.4%) before ART initiation (Table 2).

**Table 2:** Viral load and CD4 cell count, according to the presence of opportunistic infections at presentation, Mario Catarino Rivas National Hospital, January to December 2023

	Without OIs		With OIs		Total	
	N=79		N=31		N=110	
	Number (n)	Percentage (%)	Number (n)	Percentage (%)	Number (n)	Percentage (%)
Viral load (copies/ml)						
<1,000	10	12.7	2	6.5	12	10.9
1,000-100,000	50	63.3	8	25.8	58	52.7
>100,000	15	19.0	17	54.8	32	29.1
No reagent	4	5.1	4	12.9	8	7.3
CD4 cell count (cell/mm^3^)						
<200	11	13.9	21	67.7	32	29.1
200-500	39	49.4	5	16.1	44	40.0
>500	20	25.3	4	12.9	24	21.8
No reagent	9	11.4	1	3.2	10	9.1

In terms of clinical stage, one (0.9%) patient was classified as Category N, 74 (67.3%) as Category A, four (3.6%) as Category B, and 31 (28.2%) as Category C. Only two (1.8%) patients were using PrEP. Of 110 patients, 88 (80%) had no comorbidities before their HIV diagnosis. The most common comorbidities were arterial hypertension 5/110 (4.5%), diabetes mellitus 5/110 (4.5%), dyslipidemia 3/110 (2.7%), bronchial asthma 3/110 (2.7%), and psychiatric disorders 3/110 (2.7%).

## Discussion

In the current study, opportunistic infections were diagnosed in 28.2% of patients at the time of their diagnosis of HIV infection, and in order of frequency were: cryptococcosis, candidiasis, tuberculosis, toxoplasmosis, pneumocystis, and histoplasmosis. The prevalence found in this study is comparable to the prevalence of 33.1% in a very similar study performed in a tertiary care center in Ghana [7].

These findings contrast with those of the 2016 and 2022 cohorts from the same care center. Of note, both previous studies were conducted in patients without differentiating whether they were treatment-naive and focused on data other than OI prevalence [6,8]. In 2016, the three most frequent OIs were tuberculosis, candidiasis, and toxoplasmosis. Cryptococcosis accounted for 7.5% of OI cases [6]. In 2022, in a sample of 160 patients, the most common OIs were tuberculosis, histoplasmosis, and cytomegalovirus. Here too, cryptococcosis accounted for 7.5% of the OIs described [8]. A possible cause of this change is the variable availability of laboratory reagents and imaging tests in this care center for the diagnosis of OIs.

Our results also differ from those found in the meta-analysis of OIs in low- and middle-income countries published in the Clinical Infectious Diseases journal, which reported candidiasis, tuberculosis, herpes zoster, and bacterial pneumonia as the most common OIs [9]. The two most common OIs in our study are mycosis. It is consistent with the fact that the incidence of invasive infections caused by opportunistic mycoses in Latin America seems to be increasing [10]. In sub-Saharan Africa, there is a high incidence of cryptococcosis-related deaths. For example, in Sierra Leone, the prevalence of cryptococcosis was 4.7% [11].

Clinical similarity was found with another study in Asia, where most patients were classified as Category A with a CD4+ count >500 cells/μL [12]. In another retrospective study on treatment-naive HIV patients, a baseline CD4+ count with a median of 184 cells/mm^3^ and a baseline viral load with a median of 51,194 copies/ml were found, with a high prevalence of fungal OIs [13]. Finally, in our study, most patients with at least one OI had a CD4+ count <200 cells/mm^3^, congruent with that described in previous literature [14].

Only two out of 110 patients in this study were using PrEP. This may be explained by the fact that the National Health Ministry only started providing access to PrEP in December 2022 [15], and much of our population does not know its benefits yet.

Limitations

This study was conducted at a single secondary care center during the span of a year, which may limit the generalizability of the findings to other regions. Additionally, the shortages of diagnostic resources in this care center may have influenced the observed trends.

## Conclusions

The findings of this study underscore the importance of prevention and early detection of HIV infections. The majority of patients with at least one OI had a viral load >100,000 copies/ml and a CD4+ cell count <200 cells/mm^3^. The prevalence of OIs in treatment-naïve HIV patients was 28.2%. The most frequent OIs were cryptococcosis, candidiasis, and tuberculosis. Moreover, we noted an increase in the prevalence of fungal OIs in treatment-naïve HIV patients in Honduras. Adequate allocation of resources for the diagnosis and treatment of these OIs in the country's healthcare system is paramount.

## Figures and Tables

**Figure 1:**
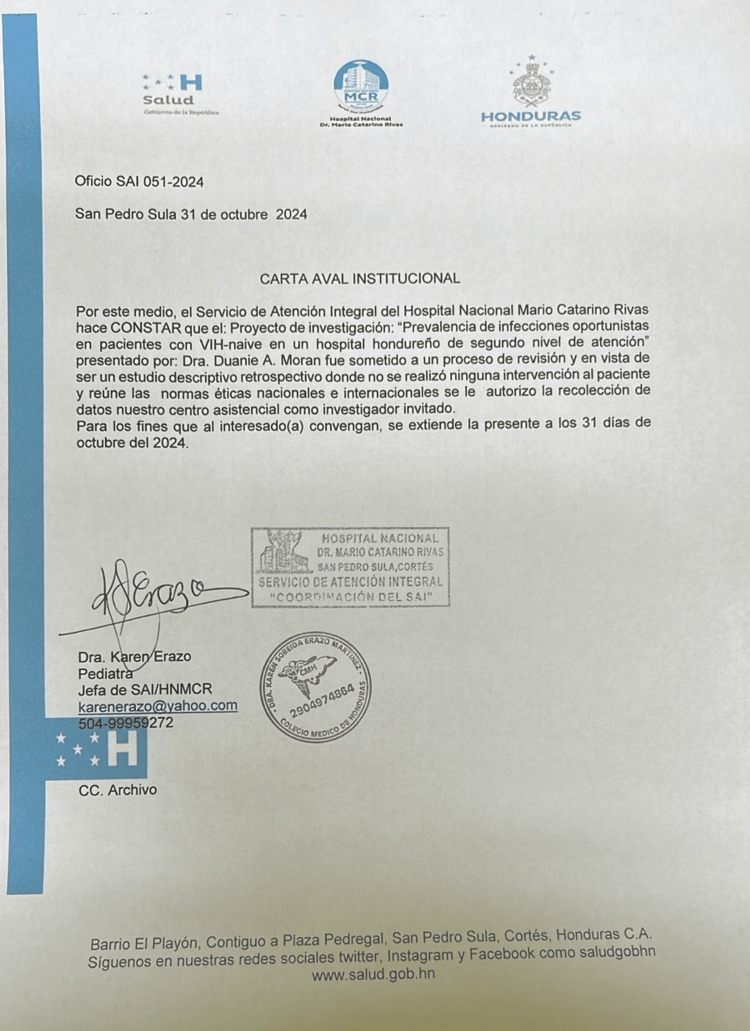
Mario Catarino Rivas National Hospital's HIV Integrated Service Protocol Approval

**Figure 2:**
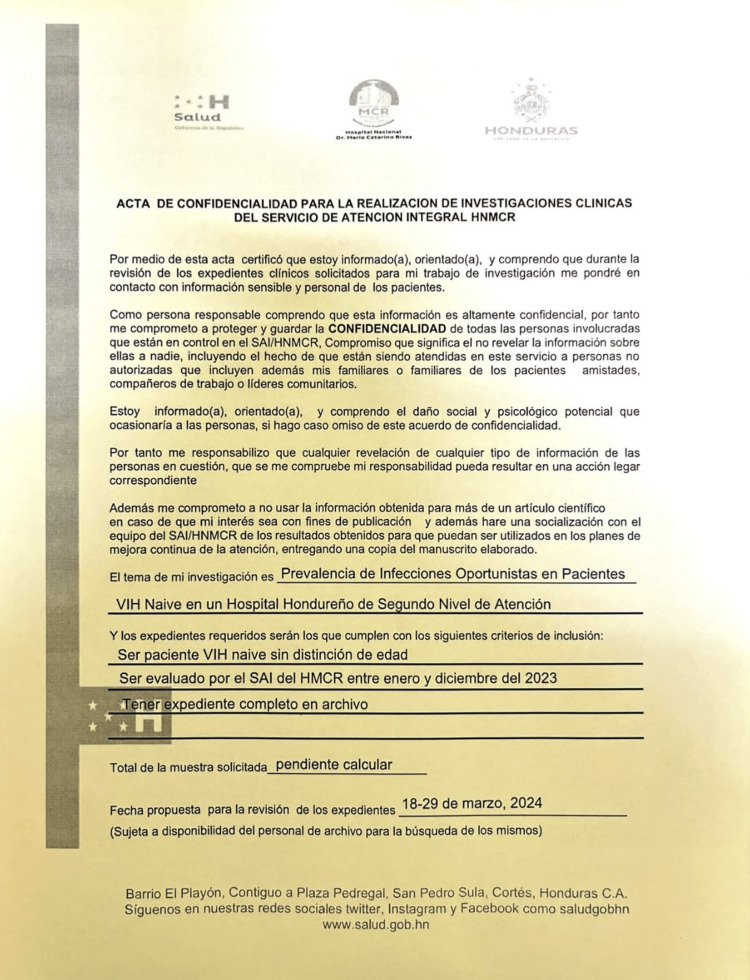
Mario Catarino Rivas National Hospital's HIV Integrated Service Confidentiality Agreement (page 1)

**Figure 3:**
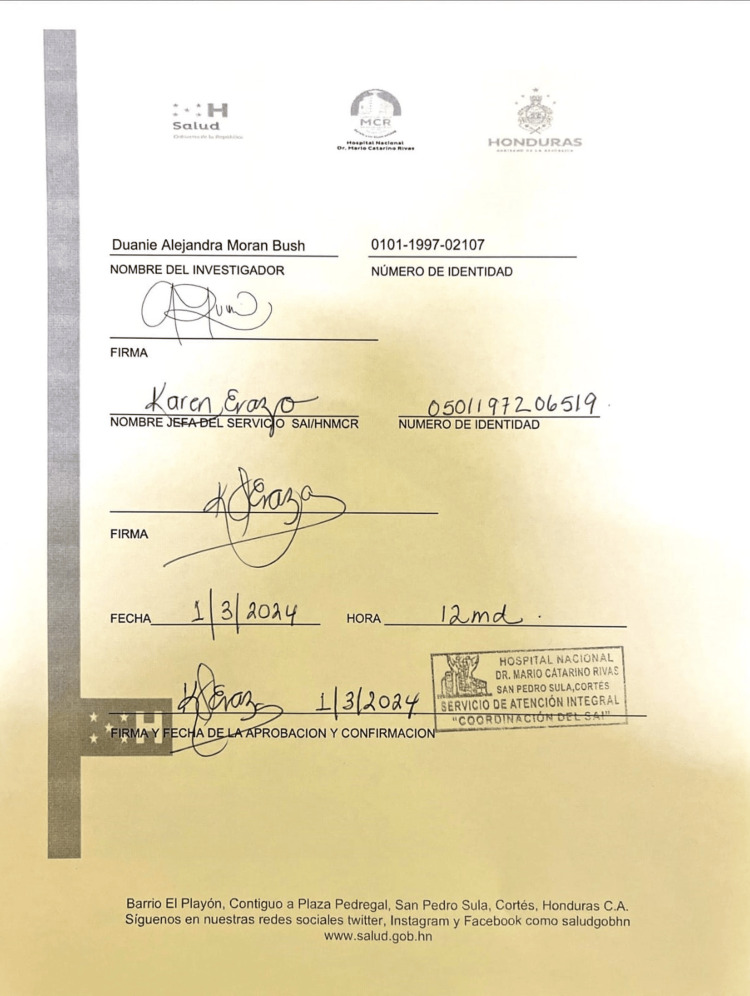
Mario Catarino Rivas National Hospital's HIV Integrated Service Confidentiality Agreement (page 2)

## Appendices

Appendix 1

Appendix 2

Appendix 3 

## References

[1] Velasco M, Torralba M, Almuedo A (2022). Document on Prevention and Treatment of Opportunistic Infections and Other Coinfections in Patients with HIV Infection (Book in Spanish). Actualización.

[2] UNAIDS_FactSheet_es.pdf UNAIDS_FactSheet_es.pdf (2024). Global HIV & AIDS Statistics: Fact sheet. https://www.unaids.org/en/resources/fact-sheet.

[3] Moylett EH, Shearer WT (2002). HIV: clinical manifestations. J Allergy Clin Immunol.

[4] Iribarren JA, Rubio R, Aguirrebengoa K (2016). Executive summary: prevention and treatment of opportunistic infections and other coinfections in HIV-infected patients: May 2015. Enferm Infecc Microbiol Clin.

[5] Núñez I, Crabtree-Ramirez B, Shepherd BE (2022). Late-onset opportunistic infections while receiving anti-retroviral therapy in Latin America: burden and risk factors. Int J Infect Dis.

[6] Lujan K, Erazo K (2019). Diagnostic limitations of opportunistic infections in patients with HIV (Article in Spanish). Rev Científica Esc Univ Las Cienc Salud.

[7] Puplampu P, Asafu-Adjaye O, Harrison M, Tetteh J, Ganu VJ (2024). Opportunistic Infections among newly diagnosed HIV patients in the largest tertiary facility in Ghana. Ann Glob Health.

[8] Carballo KB, Vides MG, Sierra JM, Erazo KE, López RM (2022). Proportion of opportunistic infections and risk factors associated with their appearance in patients with HIV (Article in Spanish). Rev Científica Esc Univ Las Cienc Salud.

[9] Low A, Gavriilidis G, Larke N (2016). Incidence of opportunistic infections and the impact of antiretroviral therapy among HIV-infected adults in low- and middle-income countries: a systematic review and meta-analysis. Clin Infect Dis.

[10] Nucci M, Queiroz-Telles F, Tobón AM, Restrepo A, Colombo AL (2010). Epidemiology of opportunistic fungal infections in Latin America. Clin Infect Dis.

[11] Lakoh S, Rickman H, Sesay M (2020). Prevalence and mortality of cryptococcal disease in adults with advanced HIV in an urban tertiary hospital in Sierra Leone: a prospective study. BMC Infect Dis.

[12] Gangcuangco LM, Sawada I, Tsuchiya N (2017). Regional differences in the prevalence of major opportunistic infections among antiretroviral-naïve human immunodeficiency virus patients in Japan, Northern Thailand, Northern Vietnam, and the Philippines. Am J Trop Med Hyg.

[13] Oladele R, Ogunsola F, Akanmu A, Stocking K, Denning DW, Govender N (2020). Opportunistic fungal infections in persons living with advanced HIV disease in Lagos, Nigeria; a 12-year retrospective study. Afr Health Sci.

[14] Justiz Vaillant AA, Naik R (2024). HIV-1-Associated Opportunistic Infections. StatPearls [Internet].

[15] User S (2024). Ministry of Health launches the PrEP strategy for HIV (Website in Spanish). https://sshome.salud.gob.hn/index.php/component/k2/item/360-secretaria-de-salud-lanza-la-estrategia-prep-al-vih..

